# Supplementary material for: Resolving the full spectrum of human genome variation using Linked-Reads
Source: Genome Res. 2019 Apr;29(4):635–45. doi: 10.1101/gr.234443.118 (PMC6442396; doi:10.1101/gr.234443.118)
Supplement: Supplemental Material [file supp_gr.234443.118_Supplemental_longranger-2.2.zip › longranger-2.2.tar.bz2!/lr2.2-public/lib/stan/cmdstan/stan/doc/api/html/agrad_2rev_2functions_2bessel__second__kind_8hpp_source.html]

Stan: src/stan/agrad/rev/functions/bessel\_second\_kind.hpp Source File


|  |  |
| --- | --- |
|  | Stan  2.5.0  probability, sampling & optimization |


- Main Page
- Related Pages
- Namespaces
- Classes
- Files

- File List
- File Members

All Classes Namespaces Files Functions Variables Typedefs Enumerations Enumerator Friends Macros Pages

- src
- stan
- agrad
- rev
- functions

bessel\_second\_kind.hpp

Go to the documentation of this file.

1 #ifndef STAN\_\_AGRAD\_\_REV\_\_FUNCTIONS\_\_BESSEL\_SECOND\_KIND\_HPP

2 #define STAN\_\_AGRAD\_\_REV\_\_FUNCTIONS\_\_BESSEL\_SECOND\_KIND\_HPP

3

4 #include <stan/agrad/rev/var.hpp>

5 #include <stan/agrad/rev/internal/vv\_vari.hpp>

6 #include <stan/agrad/rev/internal/vd\_vari.hpp>

7 #include <stan/agrad/rev/internal/dv\_vari.hpp>

8 #include <stan/math/constants.hpp>

9 #include <stan/math/functions/bessel\_second\_kind.hpp>

10

11 namespace stan {

12  namespace agrad {

13

14  namespace {

15

16  class bessel\_second\_kind\_dv\_vari : public op\_dv\_vari {

17  public:

18  bessel\_second\_kind\_dv\_vari(int a, vari\* bvi) :

19  op\_dv\_vari(stan::math::bessel\_second\_kind(a, bvi->val\_), a, bvi) {

20  }

21  void chain() {

22  bvi\_->adj\_ += adj\_ \* (ad\_ \* stan::math::bessel\_second\_kind(ad\_, bvi\_->val\_) / bvi\_->val\_ - stan::math::bessel\_second\_kind(ad\_ + 1, bvi\_->val\_));

23  }

24  };

25  }

26

27  inline var bessel\_second\_kind(const int& v,

28  const var& a) {

29  return var(new bessel\_second\_kind\_dv\_vari(v, a.vi\_));

30  }

31

32  }

33 }

34 #endif

dv\_vari.hpp

stan::math::bessel\_second\_kind

T2 bessel\_second\_kind(const int v, const T2 z)

**Definition:** bessel\_second\_kind.hpp:40

vd\_vari.hpp

bessel\_second\_kind.hpp

stan::agrad::var::vi\_

vari \* vi\_

Pointer to the implementation of this variable.

**Definition:** var.hpp:40

stan::agrad::bessel\_second\_kind

fvar< T > bessel\_second\_kind(int v, const fvar< T > &z)

**Definition:** bessel\_second\_kind.hpp:15

var.hpp

stan::agrad::var

Independent (input) and dependent (output) variables for gradients.

**Definition:** var.hpp:27

vv\_vari.hpp

constants.hpp


---

[ Stan Home Page ]

*© 2011–2014,
Stan Development Team.*
